# The Future of Infodemic Surveillance as Public Health Surveillance

**DOI:** 10.3201/eid2813.220696

**Published:** 2022-12

**Authors:** Howard Chiou, Christopher Voegeli, Elisabeth Wilhelm, Jessica Kolis, Kathryn Brookmeyer, Dimitri Prybylski

**Affiliations:** Centers for Disease Control and Prevention, Atlanta, Georgia, USA (H. Chiou, C. Voegeli, E. Wilhelm, J. Kolis, K. Brookmeyer, D. Prybylski);; US Public Health Service Commissioned Corps, Rockville, Maryland, USA (H. Chiou)

**Keywords:** public health surveillance, infodemic, vaccination, misinformation, disinformation, COVID-19, coronavirus disease, SARS-CoV-2, severe acute respiratory syndrome coronavirus 2, viruses, respiratory infections, zoonoses, vaccine-preventable diseases

## Abstract

Public health systems need to be able to detect and respond to infodemics (outbreaks of misinformation, disinformation, information overload, or information voids). Drawing from our experience at the US Centers for Disease Control and Prevention, the COVID-19 State of Vaccine Confidence Insight Reporting System has been created as one of the first public health infodemic surveillance systems. Key functions of infodemic surveillance systems include monitoring the information environment by person, place, and time; identifying infodemic events with digital analytics; conducting offline community-based assessments; and generating timely routine reports. Although specific considerations of several system attributes of infodemic surveillance system must be considered, infodemic surveillance systems share several similarities with traditional public health surveillance systems. Because both information and pathogens are spread more readily in an increasingly hyperconnected world, sustainable and routine systems must be created to ensure that timely interventions can be deployed for both epidemic and infodemic response.

As safe and effective COVID-19 vaccines have become more widely available, vaccine misinformation and disinformation have continued to permeate societies, often at astonishing rates. In fall 2021, nearly 2 years into the pandemic in the United States, 78% of persons believed or were unsure about >1 falsehood about COVID-19 or the vaccine (e.g., COVID-19 deaths are exaggerated or vaccines contain microchips), and 32% believed or were unsure about >4 falsehoods (*1*). Globally, rumors, stigma, and conspiracy theories about COVID-19 have been pervasive (*2*), and persons can find information about COVID-19 to be conflicting, confusing, and overwhelming (*3*).

The overabundance of information (accurate or not) that occurs during an epidemic has been referred to as an infodemic and was highlighted as a major threat by the World Health Organization (WHO) (*4*) and the US Surgeon General (*5*). We consider infodemics to include not only the rumors, falsehoods, and conspiracy theories that characterize misinformation (accidental falsehoods) and disinformation (deliberate or engineered falsehoods) but also an information overload of inaccurate or accurate information that can increase susceptibility to misinformation by increasing confusion and mistrust (*6*). Conversely, a lack of high-quality information can also lead to information voids that are rapidly filled by misinformation and disinformation (*7*). Infodemics can spread online through social media and messaging applications, or offline in conversations and through traditional media (e.g., newspapers, television, and radio). Because infodemics can be highly complex, responding to an infodemic requires collaboration across multiple disciplines, including the social sciences, communications, public health, epidemiology, data science, marketing, and clinical services.

Although the effect of infodemics on population health are challenging to measure, the COVID-19 infodemic probably had negative effects on health. Exposure to negative information or conspiracy theories about COVID-19 has been associated with lower acceptability of COVID-19 vaccination in many countries (*8*) and reduced likelihood of adherence to public health guidance (*9*). Effects of the COVID-19 infodemic also extend beyond vaccine hesitancy or delay, including promoting false treatments, creating drug shortages, and eroding trust in public health institutions and government (*2*), all of which can lead to negative effects on healthcare systems, societies, and economies (*10*).

Consequently, public health systems need to be able to detect and respond to outbreaks of misinformation, disinformation, information overload, and information voids, or any combination of these events. Responding to such outbreaks with public health action is only possible after these events are detected. Building surveillance systems is especially important for ensuring the sustainability of infodemic management activities, because responding to individual events on an ad hoc basis is resource-intensive and does not build preventive capacities for the future. Reactionary ad hoc approaches are not designed to identify harmful information before it becomes prevalent, representing a missed opportunity for organizations to preemptively debunk or “prebunk” harmful information (i.e., build resiliency and fill information voids) before it spreads. Permanent systems ensure that staffing and resource capacities are maintained over time and that preventive actions can be routinely implemented before a new emergency strikes.

The science of infodemic management is nascent, however, and the challenge of building systems can be daunting. In this article, we provide a vision for infodemic surveillance as a core public health function by highlighting our experiences using the COVID-19 State of Vaccine Confidence Insight Reporting System at the Centers for Disease Control and Prevention (CDC).

## CDC’s State of Vaccine Confidence Insight Reporting System

In response to the COVID-19 infodemic, CDC developed the COVID-19 State of Vaccine Confidence Insight Reporting System to routinely collect and analyze data on the public’s questions, concerns, frustrations, and circulating misinformation; these data have been used to produce and disseminate biweekly COVID-19 State of Vaccine Confidence Insights Reports since February 2021 (*11*). The overarching goal is to produce actionable insights that are grounded in data, theoretical frameworks, and an organizational strategy to guide communications content and intervention development. Reports are disseminated to ≈1,000 partners and publicly available on the CDC website. Intended audiences include public health professionals at local, state, federal, and international partner agencies.

The report is generated using a mixed-methods approach to synthesize multiple primary and secondary data sources, including social media, news media, search engine queries, polling data, scientific literature, and direct inquiries from the public submitted to CDC (Table). An iterative consensus-building process is used to analyze the data and identify themes, using a mixed inductive and deductive approach. Themes are grounded in the Behavioral and Social Drivers of Vaccination framework (*12*) and CDC’s COVID-19 Vaccinate with Confidence Strategy (*13*), focusing on the identification of the public’s concern in alignment with specific topical areas and behavioral and social variables believed to effect vaccine uptake. The themes also draw on the strategy’s 3 pillars to craft ways readers can take action, such as building trust, empowering healthcare personnel, engaging communities and individuals, and providing research opportunities.

**Table:** CDC COVID-19 State of Vaccine Confidence Reporting System inputs and sources*

Type and input	Frequency	Sources	Tactics for use
Social media listening and media monitoring			
Communication surveillance report	Daily on weekdays	• Google news • Meltwater • CrowdTangle • Native platform searches	• SOV analysis to identify themes • Emerging topics
Meltwater	Daily	• Facebook, Twitter, Instagram • Blogs • News media • Online forums	• SOV analysis • Emerging theme topics • Identify high reach and velocity topics
CDC’s OADC channel COVID-19 postmetrics	Weekly	• Sprout Social • Native OADC account analytics	• Analyze number of posts, topics • Success of messages, number of impressions, reach, number of engagements
OADC channel comment analysis	Daily on weekdays	• Native platform searches	• Sentiment analysis • Identify message gaps and voids
Direct reports			
CDC-INFO metrics	Weekly	• CDC-INFO inquiry line list • PR usage report	• Cross-compare PR usage with inquiry theme analysis • Sentiment analysis • Identify information gaps and voids
VTF media requests	Weekly	• Media request line list	• Leading indicator for news coverage • Identify information gaps and voids
Web metrics	Weekly	• Top pages • Google search queries • Top FAQs • Referring domains	• Identify information gaps and voids, • Identify keywords and search terms, changes in web traffic
Research			
Poll review	Weekly	• Harris, Pew Research, Gallup, and KFF polls • New data related to vaccine hesitancy	• Identify socio-behavior indicators related to motivation and intention to vaccinate
Literature review	Weekly	• PubMed, LitCovid, ProQuest Central, Altmetric • New data related to vaccine hesitancy	• Identify current vaccination intention • Identify barriers to vaccination
Third-party reports			
Tanaq social listening and media monitoring report	Weekly	• Meltwater • Sprout Social • First Draft • Native platform searches	• Trending topics • Demographic and geographic conversation monitoring
CrowdTangle content insights report	Biweekly	• Facebook	• Top pages (voices), groups • General trends and sentiment analysis • News analysis through posts
First Draft News vaccine misinformation insights report	Monthly	• Proprietary methods	• Media trends analysis • Emerging threats and data deficits • Online vaccine narratives
Project VCTR	Weekly	• Proprietary methods	• National and regional trends in negative attitudes toward vaccination • Conversations around Legislation
Virality Project	Weekly	• Proprietary methods	• Misinformation and disinformation trends related to COVID-19 vaccine

*CDC, Centers for Disease Prevention and Control; FAQ, frequently asked questions; KFF, Kaiser Family Foundation; OADC, Office of the Associate Director of Communication; PR, prepared response; SOV, share of voice; VCTR, Vaccine Communication Tracking and Response; VTF, Vaccine Task Force.

We prioritized identified themes by using a threat matrix and then classified and color-coded them by risk on the basis of reach, dissemination, and potential effect on vaccine confidence and vaccine uptake. We also characterized each theme by time and labeled it as increasing, decreasing, or stable over multiple reports. The intention of these visual markers is to support the use of these reports as an early warning system for public health action and to provide early detection of acute threats to public safety. For example, we highlighted conspiracy theories regarding ivermectin highlighted as a high-risk, increasing theme (*14*) and reported it more than a month before the CDC health alert warning of an increase in adverse effects from ivermectin misuse and overdose (*15*).

We identified a selection of themes on the basis of volume and potential effect on vaccine confidence. We then complied these themes into a regular biweekly report, which provides summary descriptions and community questions and concerns, frustrations, information needs, and circulating misinformation. We presented descriptors and exemplars of each theme alongside information voids identified and potential ways to act. The report highlights each theme with concrete examples and descriptions for the purposes of informing public health action.

The report is primarily qualitative in nature. Particularly for the purposes of intervention design, the qualitative nature of the report is critical because nuance and context must be considered. Although quantitative measures of pace of transmission (virality) or reach of a message on social media provide value in understanding how far the theme has spread, qualitative description of the identified themes and their context provides valuable information about the nuances of the theme itself and potential reasons why a particular message gained traction and became amplified. For example, understanding the community’s specific safety concerns about COVID-19 vaccines (e.g., infertility or thrombosis risks) must be combined with data that report on the prevalence of COVID-19 safety concerns more generally to enable the generation of actionable recommendations.

The first limitation of the CDC State of Vaccine Confidence Insight Reporting System is that report generation is highly labor-intensive and requires a specialized, interdisciplinary team to analyze a large amount of data on a regular basis. The report is generated at a national level, which can limit its usability for practitioners who work at a subnational or local levels. Third, comparing the pervasiveness of themes between different data sources requires a subjective lens; for example, many data sources do not have easily accessible information about reach, impressions, or number of views of individual content. Fourth, because public discourse can change rapidly, publications such as CDC’s State of Vaccine Confidence Insight Report must be published as soon as possible, but the dissemination of the findings may be delayed by organizational clearance and approval processes. Finally, as infodemic surveillance remains a nascent science, case definitions, data collection procedures, and reporting processes must be continually refined to better meet the needs of public health agencies and their partners.

## Vision and Considerations for Infodemic Surveillance Systems

The CDC State of Vaccine Confidence Insight Reporting System is an infodemic surveillance system in its infancy, particularly when compared with more established public health surveillance systems. Although few other examples of infodemic surveillance systems exist (*16*–*19*), the idea for these systems is not new. Multiple public health experts have pointed out the need for routinized infodemic data systems for the purposes of detecting and responding to infodemics (*20*–*24*). Furthermore, the system can be considered a progression of traditional social media and news monitoring because additional data are included to focus on the concerns and perceptions of the general public, and programmatic recommendations and research opportunities are generated beyond focusing on communication strategies alone (*25*,*26*). We propose 4 key functions of an infodemic surveillance system.

### Key Functions of an Infodemic Surveillance System

#### Monitoring the Information Environment by Person, Place, and Time

Identifying trends and patterns of misinformation, disinformation, information voids, perceptions, and questions of public health concern over time is critically important because the goal is to detect infodemics and respond quickly and effectively with public health action. An early warning system, for example, might detect an acute rise in misinformation that could be addressed through community engagement and targeted and tailored communications.

#### Using Digital Media Analytics to Identify Infodemic Events

Worldwide, most persons now regularly use social media or messaging apps (e.g., WhatsApp, Instagram, Facebook, Wechat, Douyin, and TikTok) (*27*), and the prevalence of health misinformation is high in online spaces (*28*). Data collection systems that focus on social media, websites, and other online content may provide an opportunity to assess the incidence and prevalence of misinformation, disinformation, or information gaps, although the data are not always accessible by governments or researchers, and analysis requires specialized expertise.

#### Conducting Offline Community-Based Assessments

Ideally, infodemic surveillance systems should not rely only on analyses of digital media. Many persons lack access to the internet, even in high-income countries (e.g., 15% of persons in the United States do not own a smartphone) (*29*). Digital analytics do not capture experiences lived offline, and persons increasingly communicate using direct messaging apps that do not have data readily available for researchers (e.g., dark social media, including text messages, email, WhatsApp, or Wechat) (*30*,*31*). Offline assessments could include regular surveys, polls, focus groups, observations, or rapid qualitative assessments.

#### Generating Timely Routine Reports to be Used by the Public Health Community.

Infodemic listening data can be complex, so infodemic surveillance requires both an integrated analysis of online and offline data sources and a synthesis of quantitative and qualitative data with public health judgment. Infodemic surveillance systems must create timely reports that are usable by public health authorities to design and implement interventions rapidly in both routine and emergency settings. These reports must also be timely because the advent of the internet and social media specifically have enabled rapid communication that can quickly shift as new topics and concerns dominate the public discourse.

These 4 functions highlight important commonalities with traditional public health surveillance systems. Defined as “the ongoing systematic identification, collection, collation, analysis and interpretation of disease occurrence and public health event data, for the purposes of taking timely and robust action” (*32*), many public health surveillance systems have functions that extends beyond laboratory detection of disease. Event-based surveillance systems, for example, includes media monitoring to identify stories, rumors, or other information reported from the community for the detection of outbreaks or other events of public health importance (*33*), as found in systems like the WHO’s Epidemic Intelligence from Open Sources initiative (*34*). The Behavioral Risk Factor Surveillance System in the United States uses surveys to collect data about risk behaviors (*35*). Infodemic management systems should be considered as public health surveillance systems that similarly rely on media monitoring and surveys and share the goal of monitoring trends and patterns over time to inform timely action by public health authorities.

More important, both infodemic and traditional public health surveillance systems are reliant on epidemiologic thinking. Critics might highlight that traditional public health surveillance seeks to detect disease, whereas infodemic surveillance systems fundamentally seek to detect ideas. However, the core concepts of person, place, and time are as valuable for understanding the transmission of ideas throughout a population as they are for disease. Epidemiologic models of idea transmission have long been used in fields including the evolutionary behavioral sciences and gene-culture coevolutionary theory (*36*) and have been applied specifically for the COVID-19 pandemic (*37*,*38*).

Consequently, many of the key characteristics of a traditional public health surveillance system will apply for an infodemic surveillance system. Infodemic surveillance could be active or passive, event-based or indicator-based, and would need to be designed based on both capacity and needs of the public health authority. Traditional evaluation frameworks (e.g., simplicity, flexibility, data quality, and acceptability) for surveillance systems are also largely transferrable (*39*). In contrast to traditional public health surveillance, however, we offer a few key considerations of public health surveillance system attributes with specific applications for infodemic surveillance systems.

### Considerations of Public Health Surveillance System Attributes with Specific Applications for Infodemic Surveillance Systems

#### Case Definitions

Traditional public health surveillance relies on quantitative metrics and well-established epidemiologic concepts, such as incidence and prevalence. Although similar metrics for infodemics have been proposed (*23*), their usage is nascent and global experience limited, and many of the existing tools are borrowed from marketing for the purposes of brand management rather than for public health investigation. Further complicating this situation is the fact that, unlike traditional public health surveillance, recognizing an infodemic requires an understanding of discourse and meaning; quantitative consumption metrics of a single online post, for example, are only useful if the meaning of the post and the populations involved are also understood. Contextual information is also important in determining the degree of urgency in response. A subjective, qualitative, and interpretive lens is essential for infodemic surveillance, but integrating this subjectivity into more objective measures of the rate of spread of misinformation and disinformation needs further development to ensure their utility for health programs.

#### Timeliness

Although timeliness is an important feature for all public health surveillance systems, it is critically important for infodemic surveillance. Information can be transmitted faster than infectious diseases because information lacks an incubation period and is consumed with the click of a mouse or tap of a finger. Infodemics are highly dynamic and change rapidly, and surveillance systems must be able to detect and respond at a similar pace.

#### Resolution of Place

In descriptive epidemiology, place refers to the geographic variation of disease and may refer to either specific locations (e.g., countries or counties or hospitals) or place categories (e.g., urban or rural). Although many in public health are more comfortable with country-level data (e.g., national surveys of knowledge, attitude, and practice), surveillance systems must consider their ability to focus on specific places to ensure that the geographic level of analysis for the data outputs match the geographic level of feasible interventions. For example, if intervention resources become available for a specific region or subpopulation of interest, the ability of surveillance systems to focus on those specific regions or subpopulations would greatly help inform intervention design. However, technical limitations exist, especially because many online data sources aggregated by regions or subpopulations are not readily available. In addition, communities identified may not be geographically clustered but exist in virtual spaces where interventions may need to be implemented virtually.

#### Personnel

The interdisciplinary nature of infodemics requires expertise from multiple fields of research, including the social sciences, communications, social media marketing, and public health. Surveillance systems need to be supported by both human technical capacity (e.g., infodemic managers) and institutional capacity (e.g., budgets and organizations).

#### Information Systems

Surveillance systems often appear simple on the surface but require substantial infrastructure. Data sources need to be established, incoming data must be analyzed, and users have to receive the data to take action. Each individual step requires considerable coordination, formalized partnerships, political will, organizational infrastructure, and resources.

#### Integration and Coordination.

International standards for surveillance systems emphasize the importance of harmonizing resources and working together to use methods efficiently (*32*). Because infodemic surveillance is a relatively new activity for public health, however, new units probably need to be created on organizational charts and relationships formed between departments that previously did not exist to ensure that any data generated is acted on. Responding to an infodemic event may require new partnerships, including subnational or local public health authorities, as well as collaborations with technology sectors, media companies, and fact-checking organizations.

#### Legality, Privacy, and Ethics

Although traditional public health surveillance uses more objective diagnostic criteria, infodemic surveillance requires some subjectivity and raises ethical questions. Regarding misinformation, for example, who determines whether something is factually accurate and what policies would be applied? What is the role of public health authorities as arbiters of truth? Societal concerns regarding individual freedoms of expression must be considered, as well as the fact that information often changes rapidly and what is considered factually accurate may change over time. Additional considerations exist concerning the collection of private data or data that persons perceive as private. For example, social media has made it easy to join private groups and follow individual persons who might not make their social media posts publicly available, but there are ethical considerations when doing this while not presenting oneself as a member of a public health organization.

Although those key considerations require careful deliberation when new systems are established, none are insurmountable. In fact, the same considerations are also present in traditional public health surveillance systems, although the nuances and importance of each consideration might be different in the context of infodemic surveillance. Those considerations are critically important to ensure that infodemic surveillance systems can serve as early warning systems for public health response. Within the pandemic setting, potential responses may include not only countering misinformation and crisis communication but also fighting stigma, addressing mental health, and providing psychological preparedness (*40*). Early detection and response is especially important to address inequities in the public health response and help minimize disparities as much as possible; for example, addressing misperceptions on whether undocumented persons are eligible for vaccinations or testing (*41*). These actions can only be taken, however, after an infodemic has been detected.

Although infodemic surveillance activities may be intensified during a pandemic or outbreak response, infodemic surveillance systems would be equally important in routine nonemergency settings. Infodemics affect health behaviors outside of vaccines and infectious diseases, including nutrition, cancer, and diabetes (*42*). Misinformation about e-cigarettes and vaping products circulating misinformation on social media channels popular with teens, for example, has strongly affected teenagers (*43*). Tracking and understanding these infodemics through routine infodemic surveillance systems is a critical first step toward designing interventions to promote population health. By building trust and information literacy, routine infodemic surveillance systems can potentially prevent severe infodemics that might accompany future outbreaks and emergencies.

## Conclusions and Opportunities

Based on the experience of developing and deploying the CDC State of Vaccine Confidence Reporting System, this article presents a vision for the future of infodemic surveillance systems. Although there are many similarities to traditional public health surveillance systems, we have highlighted several key considerations that require careful deliberation when establishing and evaluating infodemic surveillance systems. Evaluation is particularly important to establish scientific rigor for infodemic surveillance systems and further develop evidence-based practices within infodemic management.

It is also important to recognize that the fundamental idea of infodemic surveillance systems is not new within public health. The WHO Technical Guidelines for Integrated Disease Surveillance and Response in the WHO African Region, for example, includes language highlighting the importance of understanding public perceptions and deploying surveys, interviews, and social media monitoring (*32*). The WHO Joint External Evaluation tool, used regularly by countries to assess public health capacity, includes a risk communication technical area where countries receive maximum scores for the presence of a “strong system for listening and rumour management on a permanent basis which is integrated into the decision-making and response actions” (*44*). Communication and media monitoring have also been previously conducted in outbreak settings (*25*,*26*).

Despite those efforts, however, such activities are typically not perceived as part of routine public health surveillance. Although we have highlighted the differences between traditional and infodemic surveillance, their similarities greatly outweigh the differences. As sharing both information and pathogens spreads more readily in an increasingly hyperconnected world, sustainable and routine systems must be created to ensure that timely interventions can be deployed for both epidemic and infodemic response.
